# Comparison of Miller laryngoscope and UEScope videolaryngoscope for endotracheal intubation in four pediatric airway scenarios: a randomized, crossover simulation trial

**DOI:** 10.1007/s00431-019-03375-y

**Published:** 2019-04-11

**Authors:** Jacek Smereka, Marcin Madziala, Dominika Dunder, Elzbieta Makomaska-Szaroszyk, Lukasz Szarpak

**Affiliations:** 1 Department of Emergency Medical Service, Wroclaw Medical University, Wroclaw, Poland; 2 Lazarski University, 43 Swieradowska Str., 02-662 Warsaw, Poland

**Keywords:** Cardiopulmonary resuscitation, Endotracheal intubation, Pediatric, Physician, Videolaryngoscopy

## Abstract

With different videolaryngoscopes for pediatric patients available, UEScope can be used in all age groups. The aim of this study was to compare the Miller laryngoscope and UEScope in pediatric intubation by paramedics in different scenarios. Overall, 93 paramedics with no experience in pediatric intubation or videolaryngoscopy performed endotracheal intubation in scenarios: (A) normal airway without chest compressions, (B) difficult airway without chest compressions, (C) normal airway with uninterrupted chest compressions, (D) difficult airway with uninterrupted chest compressions. Scenario A. Total intubation success with both laryngoscopes: 100%. First-attempt success: 100% for UEScope, 96.8% for Miller. Median intubation time for UEScope: 13 s [IQR, 12.5–17], statistically significantly lower than for Miller: 14 s [IQR, 12–19.5] (*p* = 0.044). Scenario B. Total efficacy: 81.7% for Miller, 100% for UEScope (*p* = 0.012). First-attempt success: 48.4% for Miller, 87.1% for UEScope (*p* = 0.001). Median intubation time: 27 s [IQR, 21–33] with Miller, 15 s [IQR, 14–21] with UEScope (*p* = 0.001). Scenario C. Total efficiency: 91.4% with Miller, 100% with UEScope (*p* = 0.018); first-attempt success: 67.7 vs. 90.3% (*p* = 0.003), respectively. Intubation time: 21 s [IQR, 18–28] for Miller, 15 s [IQR, 12–19.5] for UEScope. Scenario D. Total efficiency: 65.6% with Miller, 98.9% with UEScope (*p* < 0.001); first-attempt success: 29.1 vs. 72% (*p* = 0.001), respectively. Intubation time: 38 s [IQR, 23–46] for Miller, 21 s [IQR, 17–25.5] for UEScope.

*Conclusion*: In pediatric normal airway without chest compressions, UEScope is comparable with Miller. In difficult pediatric airways without chest compressions, UEScope offers better first-attempt success, shorted median intubation time, and improved glottic visualization. With uninterrupted chest compressions in normal or difficult airway, UEScope provides a higher first-attempt success, a shorter median intubation time, and a better glottic visualization than Miller laryngoscope.

**Table:** 

**What is Known:** • *Endotracheal intubation is the gold standard for adult and children airway management.* • *More than two direct laryngoscopy attempts in children with difficult airways are associated with a high failure rate and increased incidence of severe complications.*	
**What is New:** • *In difficult pediatric airways with or without chest compressions, UEScope in inexperienced providers in simulated settings provides better first-attempt efficiency, median intubation time, and glottic visualization.*

## Introduction

In pediatric acute care settings, endotracheal intubation is the golden standard for securing the airway in situations where the provider is unable to ventilate the patient adequately with a bag-and-mask or by a supraglottic airway device, or if an open airway is compromised. Cases of inability to perform bag-mask ventilation or to obtain correct placement of supraglottic airway devices, especially among non-anesthesiological personnel or in patients with difficult airway, emphasize the role of videolaryngoscopes [3]. The pediatric and adult airway anatomy and physiology differ, and medical personnel experience in direct pediatric intubation can be limited [23].

A complication of failed endotracheal intubation is hypoxemia [4]. A way to reduce the potential complications, including airway edema, is to decrease the number of total intubation attempts and the procedure duration, which is possible with better or alternative intubation technique, including videolaryngoscopy [14, 18]. Fiadjoe et al. suggest that more than two direct laryngoscopy attempts in children with difficult airway are associated with a high rate of failure and severe complications [4]. Moreover, direct pediatric laryngoscopy is bound with significant interruptions in chest compressions [2].

Videolaryngoscopy offers a better viewing angle and less traumatic intubation, can reduce the complication rate [15, 17, 30], and supports intubation training [13].

Many different videolaryngoscopes for pediatric patients are available, but not all can be used in all age groups [23, 30]. UEScope is a portable videolaryngoscope [29] invented in 2010, equipped with several blades for different age groups. Its specific feature is the angulated blade [29]. The technique for introducing UEScope does not demand tongue sweep. UEScope has been tested in various clinical conditions and most of the studies were performed in Chinese pediatric patients [23, 24]. The results, though inconsistent, suggest that UEScope is not inferior to direct laryngoscopy. As is true for many videolaryngoscopes, the UEScope learning curve is better than that for direct laryngoscopy. Owing to the slim design and low weight, UEScope is easy to manipulate [29].

Videolaryngoscopy with the use of numerous devices in difficult pediatric airway with continuous chest compressions has been tested in several studies. Comparing GlideScope and direct laryngoscopy in a pediatric simulator by novice physicians, Rabiner et al. found that GlideScope did not improve time to intubation or intubation success rates in normal or difficult airway scenarios [16]. In turn, in a study by Szarpak et al. concerning pediatric endotracheal intubation with chest compressions, the first-attempt intubation and overall intubation success were better with GlideScope than with Miller direct laryngoscopy [25]. TruView offered better intubation conditions than Macintosh in a pediatric manikin scenario with chest compressions with and without cervical stabilization [24].

The UEScope was selected because it is a portable device with angulated or Miller’s blade which can be used in all age group and the results concerning effectiveness in endotracheal intubation obtained so far were inconsistent.

The aim of the study was to compare the Miller laryngoscope and UEScope during pediatric intubation performed by paramedics in different airway conditions.

## Methods

The study was designed as a prospective, multicenter, randomized, crossover simulation trial. It was performed in Warsaw, Wroclaw, and Poznan, Poland, between December 2017 and May 2018, in accordance with the Consolidated Standards of Reporting Trials (CONSORT) [17]. The protocol was approved by the Institutional Review Board of the Polish Society of Disaster Medicine (approval No. 21.11.2017.IRB).

### Participants

The study included 93 paramedics participating in Pediatric Advance Life Support courses based on the American Heart Association guidelines. Voluntary written informed consent was obtained from all participants. The inclusion criteria were the following: (1) not having performed more than 100 clinical adult intubations with direct laryngoscopy and no experience in clinical pediatric intubation; (2) no experience in clinical or experimental training in videolaryngoscopy [24]. Wrist or back injury up to 1 month before the study was the exclusion criterion.

### Scenarios

To simulate endotracheal tube immediate airway management conditions in a 5-year-old child, the Pediatric HAL® S3005 simulator was used (Gaumard® Scientific, Miami, USA). The participants performed intubation in four scenarios:

1. Scenario A: normal airway without chest compressions.
2. Scenario B: difficult airway without chest compressions. The simulator control software inflated the tongue to simulate conditions of Mallampati scale grade III [18].
3. Scenario C: normal airway with uninterrupted chest compressions. To standardize the difficulties resulting from chest compressions, the Corpuls system (GS Elektromedizinische Geräte G. Stemple GmbH, Kaufering, Germany) was used to apply chest compressions (depth, 5 cm; frequency, 100 per minute).
4. Scenario D: difficult airway with uninterrupted chest compressions. As in scenario B, tongue inflation was applied. The Corpuls system performed chest compressions as in scenario C.

In all scenarios, the simulator was placed on a flat surface in a well-lit room with the head in neutral position. In both tested devices, the blade size No. 2 was used. In both tested devices, Miller’s blade was used. Endotracheal intubation was performed with a standard sealed tube of 5.5 internal diameter. A stylet was used, moistened with a lubricant, like the endotracheal tube itself. The shape of the stylet was standard and the tip of the stylet was bent to form a 90° angle. After each intubation attempt, the participant had to confirm ventilation correctness using a self-inflating bag (Ambu, Copenhagen, Denmark).

### Study protocol

**Fig. 1:**
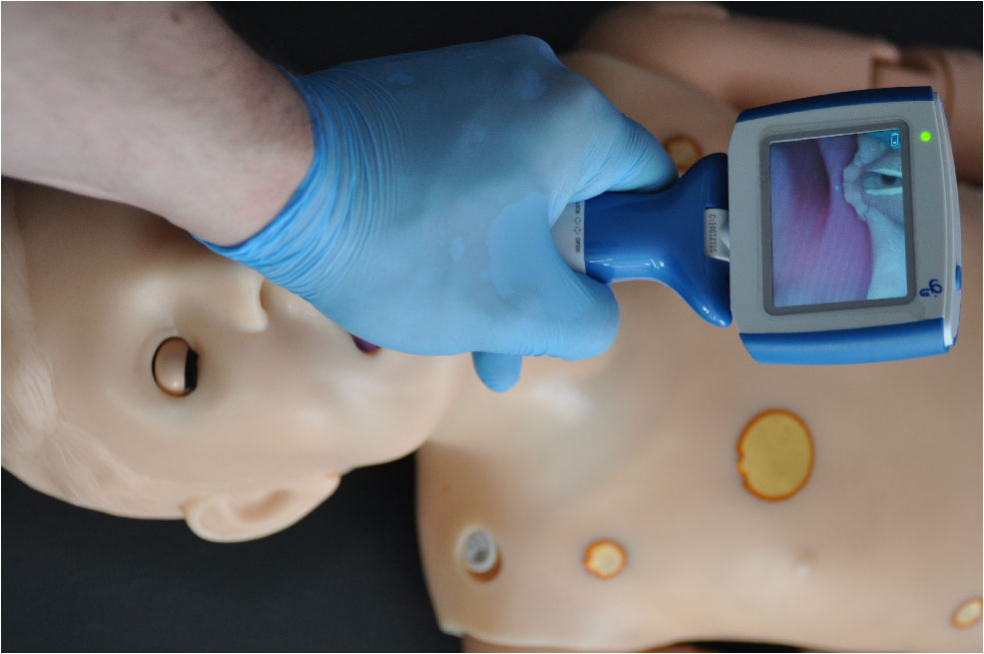
Intubation with a UEScope videolaryngoscope

Prior to the study, all paramedics participated in a 30-min training, covering the anatomy, physiology, and pathophysiology of the pediatric respiratory tract, as well as endotracheal intubation with the tested devices. The training with the Miller laryngoscope (Heine USA Ltd., Dover, USA) and UEScope (Zhejiang UE Medical Corp., Zhejiang, China) was performed by an experienced anesthesiologist (Fig. 1). Then, the participants had up to 10 min to practice intubation with the tested devices in normal adult airways with the AT Kelly Torso manikin (Laerdal, Stavanger, Norway).

One week after the training, the paramedics performed endotracheal intubation in the pediatric patient in various scenarios of simulated cardiopulmonary resuscitation using the tested devices.

**Fig. 2:**
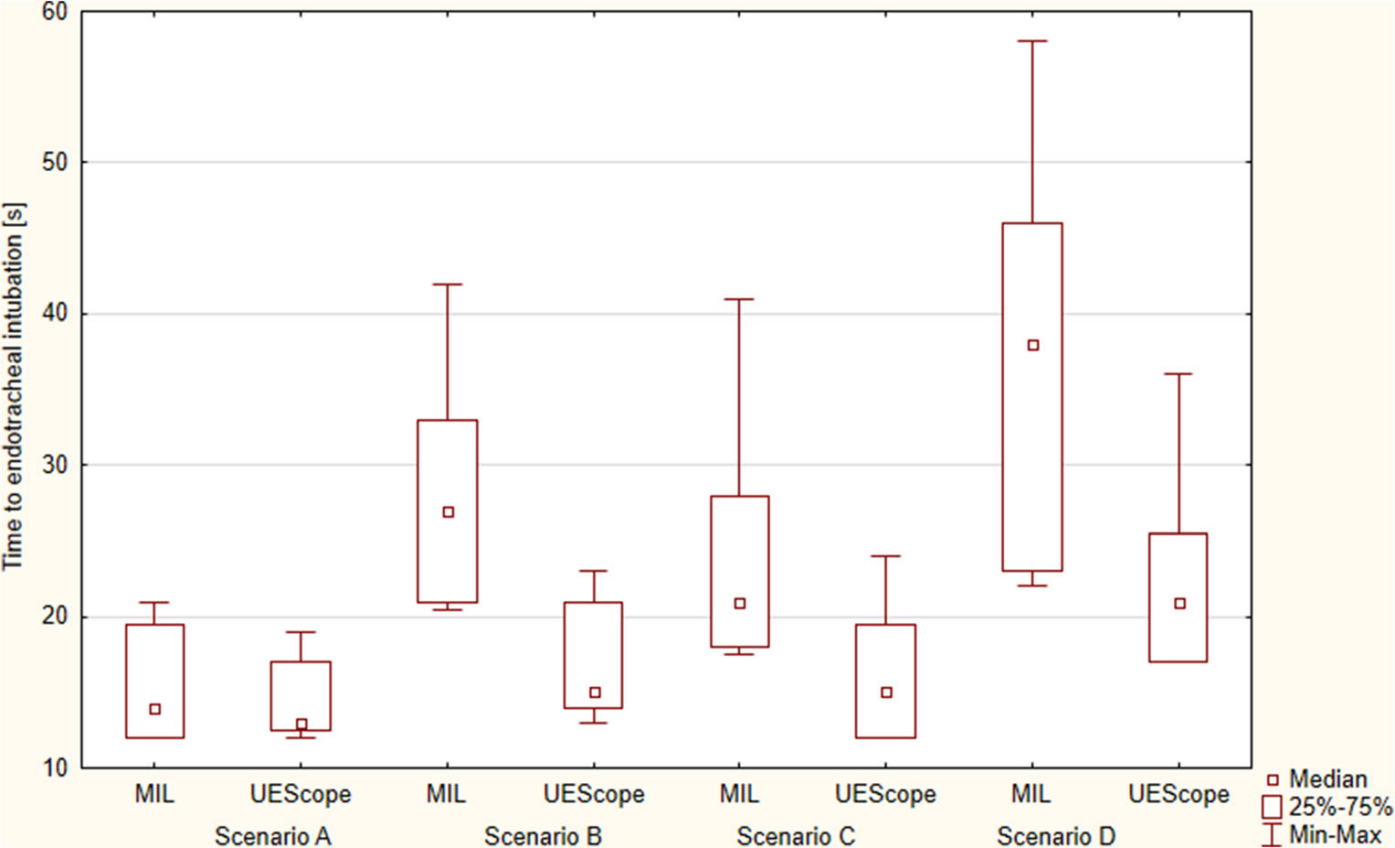
Time to intubation during the study

The order of participants and intubation methods was random: the Research Randomizer program (randomizer.org) was used. Figure 2 presents the detailed randomization procedure. Each participant had up to three attempts to intubate with each laryngoscope. Then a 10-min break was taken before the participant performed the intubation scenarios with the remaining intubation device. The paramedics were informed that the patient required immediate endotracheal intubation.

### Measurements and outcomes

The primary outcome was first intubation attempt success, defined as effective ventilation with an endotracheal tube, accompanied by a chest rise in the simulator and air flow in the lungs recorded by the simulator software. The following criteria determined failed intubation: more than three unsuccessful attempts, intubation procedure exceeding 120 s, or unrecognized esophageal intubation.

The secondary outcomes included intubation time, time to best glottic view, percentage of glottic opening and the Cormack-Lehane grade, and the ease of intubation. The intubation time was defined as the time from grasping the laryngoscope to the first attempt of effective ventilation with a self-expanding bag. The measured “time to best glottic view” was defined as “I can see the vocal cords.” The study participants assessed the glottic visibility degree: using percentage of glottic opening (POGO) [28] and the Cormack-Lehane grade [5]. A 100% POGO score indicates visualization of the entire glottic, while a 0% POGO score means no visualization of the laryngeal structures. After each intubation, the paramedics also described the ease of intubation on a scale from 1 (very easy) to 100 (very difficult).

### Sample size calculation and statistical analysis

The sample size was calculated with the G*Power 3.1 software, and the two-tailed *t* test was applied (Cohen’s *d*, 0.8; alpha error, 0.05; power, 0.95). The minimum of 61 participants turned out necessary to achieve an acceptable level of significance and power of the study; 93 were involved.

## Results

### Demographics

The study involved 93 paramedics (26 female; 27.9%) with no experience in videolaryngoscopy. All were employees of Emergency Medical Service teams. Their median age was 32 years [IQR, 29–37.5], and median work experience in emergency medicine equaled 6.5 years [IQR, 4–9].

### Scenario A: normal airway without chest compressions

The overall success with UEScope as well as with Miller was 100%. The first-attempt success was 100% for UEScope, and 96.8% for Miller (Table 1). The median intubation time for UEScope was 13 s [IQR, 12.5–17], lower than with Miller (14 s [IQR, 12–19.5]) (*p* = 0.044; Fig. 2).

**Table 1:** Study outcomes by laryngoscope devices (*n* = 93)

Outcome	Miller laryngoscope	UEScope videolaryngoscope	*p* value
Scenario A: normal airway without chest compressions			
Success of intubation attempts [%]			
1st	90 (96.8%)	93 (100%)	NS
2nd	3 (3.2%)	–	
3rd	–	–	
Overall intubation success rate [%]	93 (100%)	93 (100%)	NS
Time to endotracheal intubation [s]	14 (12–19.5)	13 (12.5–17)	0.044
Cormack and Lehane grade			
1	71 (76.3%)	93 (100%)	0.012
2	22 (24.6%)	–	
3	–	–	
4	–	–	
POGO score, 1–100	81 (75–93)	87 (85–100)	0.037
Ease of use, 1–100	18 (15–22)	15 (10–19)	0.021
Scenario B: difficult airway without chest compressions			
Success of intubation attempts [%]			
1st	45 (48.4%)	81 (87.1%)	0.001
2nd	21 (22.6%)	12 (12.9%)	
3rd	10 (10.7%)	–	
Overall intubation success rate [%]	76 (81.7%)	93 (100%)	0.012
Time to endotracheal intubation [s]	27 (21–33)	15 (14–21)	0.001
Cormack and Lehane grade			
1	–	51 (54.8%)	< 0.001
2	14 (15.1%)	39 (42%)	
3	68 (73.1%)	3 (3.2%)	
4	11 (11.8%)	–	
POGO score, 1–100	25 (23–33)	80 (74–90)	< 0.001
Ease of use, 1–100	74 (68–82)	27 (16–35)	< 0.001
Scenario C: normal airway with chest compressions			
Success of intubation attempts [%]			
1st	63 (67.7%)	84 (90.3%)	0.003
2nd	20 (21.5%)	9 (6.7%)	
3rd	2 (2.2%)	–	
Overall intubation success rate [%]	85 (91.4%)	93 (100%)	0.018
Time to endotracheal intubation [s]	21 (18–28)	15 (12–19.5)	0.005
Cormack and Lehane grade			
1	43 (46.2%)	90 (96.8%)	0.011
2	39 (41.9%)	3 (3.2%)	
3	10 (10.7%)	–	
4	–	–	
POGO score, 1–100	61 (50–72)	81 (80–98)	< 0.001
Ease of use, 1–100	39 (31–53)	17 (14–23)	< 0.001
Scenario D: difficult airway with chest compressions			
Success of intubation attempts [%]			
1st	27 (29.1%)	67 (72%)	0.001
2nd	31 (33.3%)	20 (21.5%)	
3rd	3 (3.2%)	5 (5.4%)	
Overall intubation success rate [%]	61 (65.6%)	92 (98.9%)	< 0.001
Time to endotracheal intubation [s]	38 (23–46)	21 (17–25.5)	0.004
Cormack and Lehane grade			
1	–	47 (50.5%)	< 0.001
2	7 (7.5%)	37 (39.8%)	
3	70 (75.3%)	9 (9.7%)	
4	16 (17.2%)	–	
POGO score, 1–100	21 (14–27)	75 (68–89)	< 0.001
Ease of use, 1–100	85 (77–92)	30 (28–39)	< 0.001

*NS* not statistically significant, *POGO* percentage of glottis opening

**Fig. 3:**
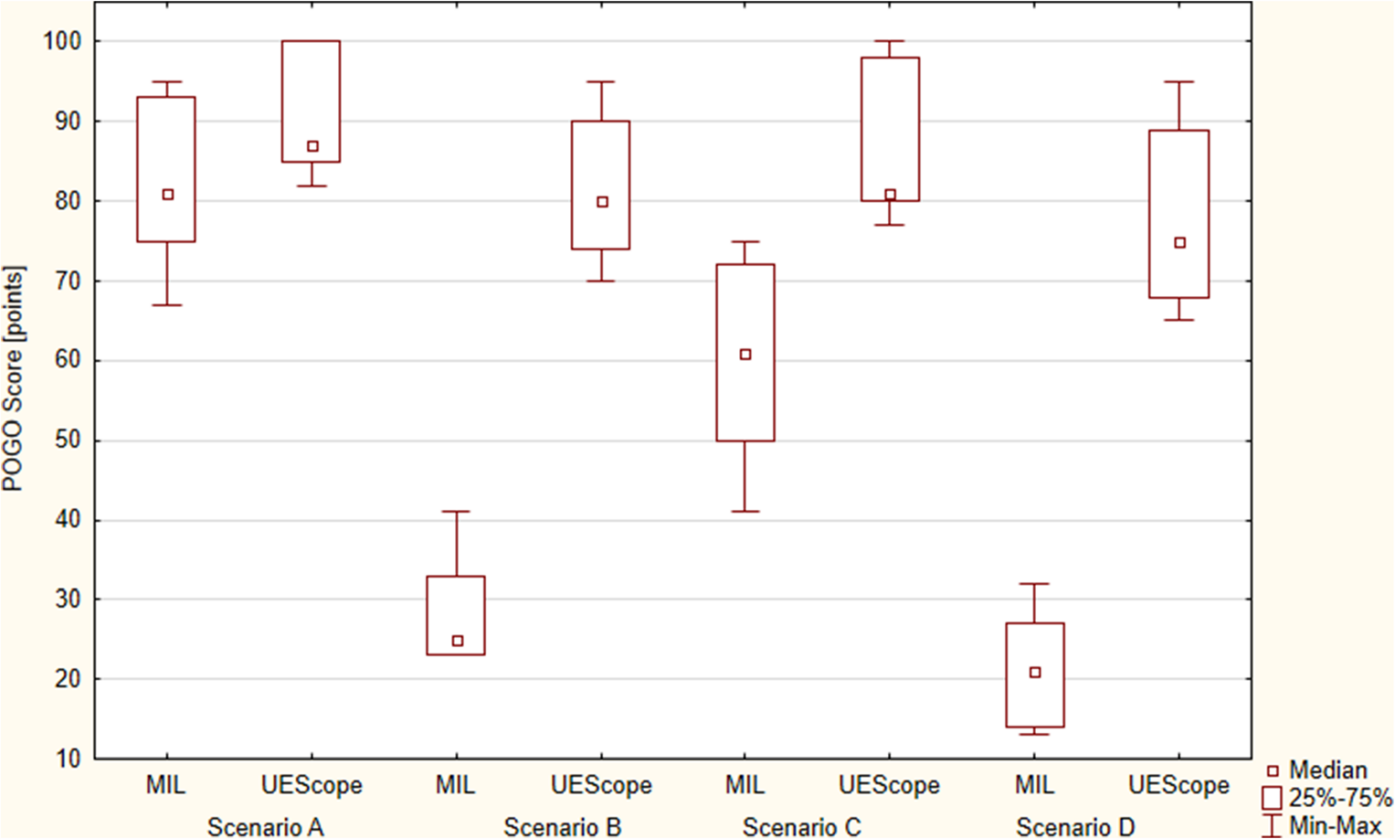
Percentage of glottic opening (POGO score)

**Fig. 4:**
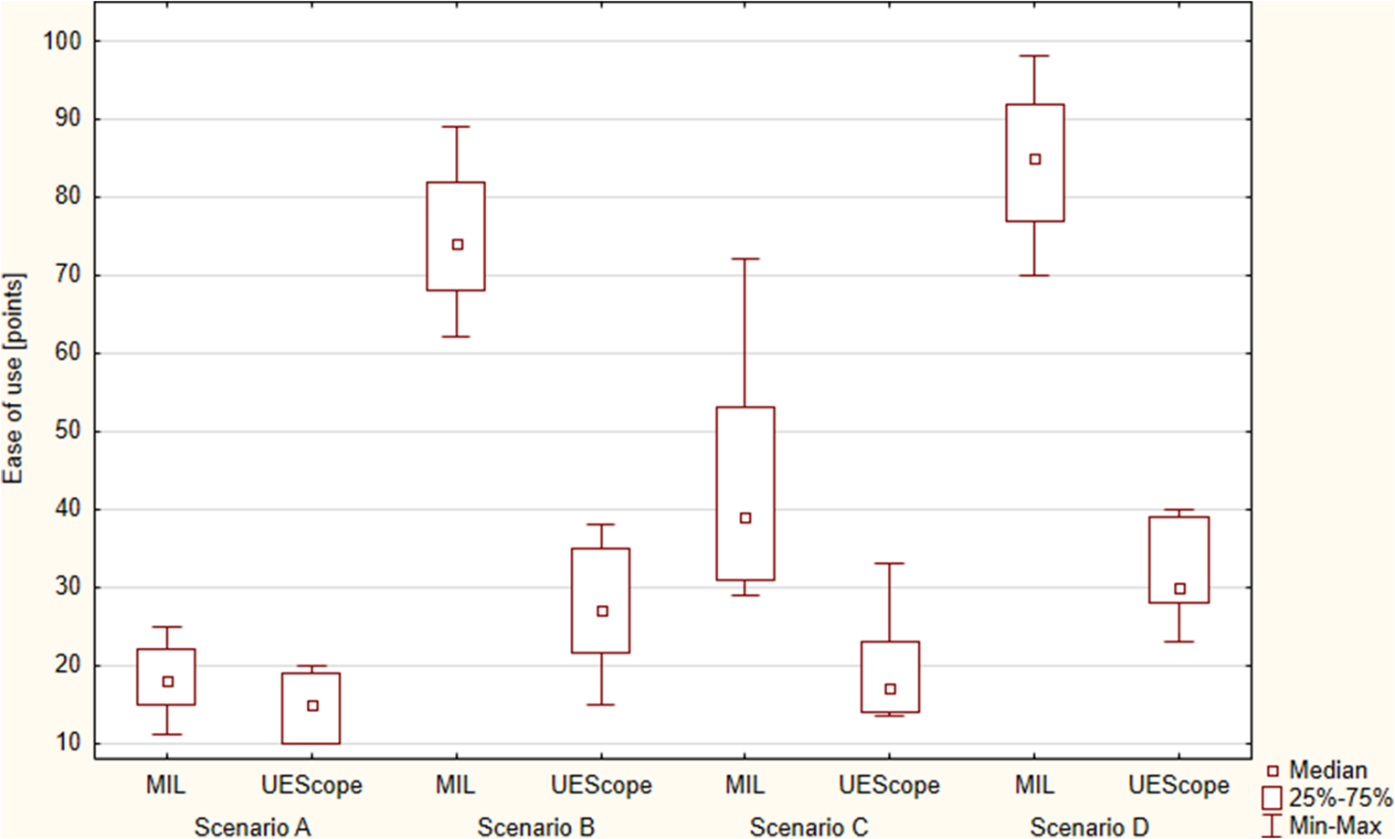
Ease of intubation with different airway scenarios

Intubation using UEScope compared with Miller was associated with a better glottic visualization based on the Cormack-Lehane scale (*p* = 0.012). The POGO score was 81% [IQR, 75–93] for Miller and 87% [IQR, 85–100] for UEScope (*p* = 0.037; Fig. 3). Intubation with UEScope proved easier in the subjective assessment as compared with Miller (15 [IQR, 10–19] vs. 18 points [IQR, 15–22]) (*p* = 0.021; Fig. 4).

### Scenario B: difficult airway without chest compressions

The total endotracheal intubation efficacy with Miller and UEScope varied and equaled 81.7 vs. 100%, respectively (*p* = 0.012). Successful endotracheal intubation on first-attempt success was 48.4% for Miller and 87.1% for UEScope (*p* = 0.001; Table 1). The median intubation time was 27 s [IQR, 21–33] for Miller and 15 s [IQR, 14–21] for UEScope (*p* = 0.001; Fig. 2). The degree of glottic visualization based on the Cormack-Lehane scale proved better for UEScope (*p* < 0.001). The POGO score was 25% [IQR, 23–33] for Miller and 80% [IQR, 74–90] for UEScope (*p* < 0.001; Fig. 3). The ease of use amounted to 74 points [IQR, 68–82] for Miller and 27 points [IQR, 16–25] for UEScope (*p* < 0.001; Fig. 4).

### Scenario C: normal airway with uninterrupted chest compressions

The overall success was 91.4% with Miller and 100% with UEScope (*p* = 0.018), with the successful endotracheal intubation on first-attempt success 67.7 vs. 90.3%, respectively (*p* = 0.003; Table 1). The intubation time was 21 s [IQR, 18–28] for Miller and 15 s [IQR, 12–19.5] for UEScope. The UEScope procedure was statistically significantly shorter than that with Miller (*p* = 0.005; Fig. 2). Glottic visualization with UEScope compared with Miller was also statistically significantly better (*p* = 0.011). The POGO score equaled 61% [IQR, 50–72] for Miller and 81% [IQR, 80–98] for UEScope (*p* < 0.001; Fig. 3). The intubation ease with UEScope was 17 points [IQR, 14–23], statistically significantly better than that with Miller (39 points [IQR, 31–53]) (*p* < 0.001; Fig. 4).

### Scenario D: difficult airway with uninterrupted chest compressions

The intubation efficiency was higher for UEScope compared with Miller both overall (98.9 vs. 65.6%; *p* < 0.001) and for the first attempt (72 vs. 29.1%; *p* = 0.001). UEScope proved superior to Miller also with regard to the procedure duration (21 [IQR, 17–25.5] vs. 38 s [IQR, 23–46]; *p* = 0.004; Fig. 2), glottic visualization based on the Cormack-Lehane scale (*p* < 0.001), POGO score (75% [IQR, 68–89] vs. 21% [IQR, 14–27]; *p* < 0.001; Fig. 3), and ease of use (30 [IQR, 28–39] vs. 85 points [IQR, 77–92]; *p* < 0.001; Fig. 4).

## Discussion

The use of videolaryngoscopy in normal and difficult airway has been widely tested in adults. However, videolaryngoscopes are still not regarded as the standard first-attempt devices for normal airway intubation in children [6, 10, 22]. Their application depends on the clinical settings and the intubator’s experience. Many studies have been carried out on the role of videolaryngoscopy in normal and difficult airway in pediatric patients [12, 18, 23, 24, 30, 32]. Some suggest that videolaryngoscopes offer no benefit over direct laryngoscopy performed by emergency department personnel with regard to the rate of first-pass intubation success, complications, or successful intubation [3]. Other studies reveal advantages of videolaryngoscopy in children, especially with difficult airway [9, 16, 20, 24].

In our study in normal airway without chest compressions, the median intubation time was comparable, and the differences were statically significant although not relevant from a practical point of view. In difficult airway without chest compressions, the total endotracheal intubation efficacy and median intubation time using Miller and UEScope varied, and the differences were statistically and practically important. In normal airway with uninterrupted chest compressions, those differences were also statistically important. The biggest differences were found in difficult airway with uninterrupted chest; they were statistically significant and noticeable practical significance.

UEScope has been tested in different clinical conditions in adult and pediatric patients, generally Chinese; the role of UEScope for non-Chinese patients is raised [7, 8, 21, 22, 31, 32], with the consideration of potential differences in airway anatomy. These studies were published mainly in Chinese, and their results are available thanks to publications by Xue et al. [29, 30].

Most studies concerning videolaryngoscopes in pediatric patients analyzed normal airway [19]. We investigated various situations, including difficult airway and intubation accompanied by continuous chest compressions.

Although inexperienced providers are not expected to perform pediatric intubation in their daily practice routinely, comparing personnel more experienced in direct laryngoscopy than in the use of videolaryngoscopy would be a source of methodological bias.

Wan [24] proved a better glottic view, a higher first-attempt success rate, and shorter intubation time with UEScope than with Macintosh in normal airway pediatric patients. In our simulation, endotracheal tube placement was equal in UEScope and direct laryngoscopy groups, but still the UEScope intubation time turned out shorter than that with direct laryngoscopy. The differences can be explained by the assumed definitions of time to intubation and the clinical vs. simulation setting.

In a study by Jiang and Jin [8] among 200 children with normal airway, videolaryngoscopy improved the laryngeal view and the success rate compared with Macintosh, but the intubation time was similar. We found a shorter intubation time in the difficult airway scenario, comparable with that in the clinical study by Jiang and Jin.

UEScope was also compared with direct laryngoscopy for endotracheal intubation in neonates for planned surgery. Its first-attempt success was non-inferior or better and the intubation time was shorter [1, 26, 27, 31]. The size of the groups in these studies was limited and the intubator’s experience; this can be the reason for the first-attempt success inconsistency.

## Limitations and strengths

One of the limitations is that the study was performed with a pediatric simulator. However, medical simulation allows for multiple procedures with no potential harm to the patient, and for standardization of conditions [11, 21]. Secondly, the study group was limited to paramedics. Although being inexperienced intubators, they work in emergency situations and face pediatric patients, including those requiring advanced airway management. The third limitation is the shorter learning curve and better glottic visualization with the use of videolaryngoscopy comparing to direct laryngoscopy.

The study strengths include the randomization, crossover design, comparison of various scenarios, and advanced research methods.

## Conclusions

In pediatric normal airway without chest compressions, UEScope is comparable with Miller. In difficult pediatric airways without chest compressions, UEScope offers better first-attempt success, shorted median intubation time, and improved glottic visualization. With uninterrupted chest compressions in normal or difficult airway, UEScope provides a higher first-attempt success, a shorter median intubation time, and a better glottic visualization than Miller laryngoscope.

## Abbreviations

CONSORT: Consolidated Standards of Reporting Trials
POGO: percentage of glottic opening
